# Diagnosis of Small Unruptured Intracranial Aneurysms: Comparison of 7 T versus 3 T MRI

**DOI:** 10.1007/s00062-023-01282-2

**Published:** 2023-03-31

**Authors:** Piotr Radojewski, Tomas Dobrocky, Mattia Branca, William Almiri, Manuel Correia, Andreas Raabe, David Bervini, Jan Gralla, Roland Wiest, Pasquale Mordasini

**Affiliations:** 1 Institute of Diagnostic and Interventional Neuroradiology, Bern University Hospital, Inselspital, University of Bern, Freiburgstrasse 18, 3010 Bern, Switzerland; 2 Translational Imaging Center, sitem-insel, Bern, Switzerland; 3 CTU Bern, University of Bern, Bern, Switzerland; 4 Hospital de Santa Maria-Centro Hospitalar Lisboa Norte, Lisbon, Portugal; 5 Department of Neurosurgery, Bern University Hospital, Inselspital, University of Bern, Bern, Switzerland; 6 Netzwerk Radiologie, Kantonsspital St. Gallen, St. Gallen, Switzerland

**Keywords:** 7 T magnetic resonance imaging, Ultra-high-field magnetic resonance imaging, UHF magnetic resonance imaging, Unruptured intracranial aneurysm, Aneurysm

## Abstract

**Purpose:**

Differentiating normal anatomical variants such as an infundibulum or a vascular loop from true intracranial aneurysms is crucial for patient management. We hypothesize that high-resolution 7 T magnetic resonance imaging (MRI) improves the detection and characterization of normal anatomical variants that may otherwise be misdiagnosed as small unruptured aneurysms.

**Methods:**

This is a retrospective, single-center study. All patients were scanned on a clinically approved 7 T MRI scanner and on a 3 T scanner. Image analysis was performed independently by three neuroradiologists blinded to clinical information. The presence of an unruptured intracranial aneurysm (UIA) and level of diagnostic certainty were assessed and the interrater agreement was calculated. If an aneurysm was present, the anatomic location and shape were recorded and compared.

**Results:**

In total, 53 patients with equivocal cerebrovascular findings on 1.5 T or 3 T MRI referred for a 7T MRI examination were included. Aneurysms were suspected in 42 patients examined at 3 T and in 23 patients at 7 T (rate difference 36%, 95% confidence interval, CI, 19–53%, *p*-value < 0.001). Major disagreement between the field strengths was observed in the A1 segment of anterior cerebral artery/anterior communicating artery (A1/ACOM) complex. The interrater agreement among the readers on the presence of an aneurysm on 7 T MRI was higher than that for 3 T MRI (0.925, 95% CI 0.866–0.983 vs. 0.786, 95% CI 0.700–0.873).

**Conclusion:**

Our analysis demonstrates a significantly higher interrater agreement and improved diagnostic certainty when small intracranial aneurysms are visualized on 7 T MRI compared to 3 T. In a selected patient cohort, clinical implementation of 7 T MRI may help to establish the definitive diagnosis and thus have a beneficial impact on patient management.

**Supplementary Information:**

The online version of this article (10.1007/s00062-023-01282-2) contains supplementary material, which is available to authorized users.

## Introduction

Equivocal neurovascular findings are a common challenge for radiologists [1, 2]; however, differentiating normal anatomical variants of brain vasculature, such as an infundibulum or a vascular loop, from true intracranial aneurysms is crucial for patient management. As well as accurate diagnosis, avoiding unnecessary repeat follow-up examinations or invasive imaging studies is essential and influences both healthcare expenses and patient comfort [3–6]. False positive findings may not only result in unnecessary psychological distress for the patient but also impose a burden on the healthcare system [7]. A recent study suggested a potential to overestimate the presence of small unruptured intracranial aneurysms (UIA) on 3 Tesla (3 T) MRI [8]. Since 2017, clinical ultrahigh field strength (UHF) 7 T MRI has been cleared for clinical applications [9]. Because of the improvement of the signal-to-noise ratio, it provides higher image resolution, and may assist in differentiating benign vascular variants from true UIAs [10, 11]. This could reduce the rate of false positive findings and have a beneficial influence on patient management. We now extended the previously presented cohort [8] and performed independent readings as opposed to the previous analysis focusing on clinical reports only. The main objective of the present study was to investigate whether the use of 7 T MRI in the clinical work-up can reduce the rate of false positive findings. We also compared the diagnostic certainty of 7 T MRI with 3 T MRI in the assessment of ambiguous neurovascular findings, such as an infundibulum or a vascular loop versus true small intracranial aneurysms. We hypothesized that high-resolution UHF MRI improves the detection rate of normal anatomical variants that may otherwise be misdiagnosed as small unruptured aneurysms.

## Methods

### Data Collection

We conducted a retrospective, single-center study which included patients examined between October 2019 and October 2021 at a tertiary care center. An expert neurovascular board comprising interventional neuroradiologists and vascular neurosurgeons reviewed all patients with equivocal cerebrovascular findings on standard brain MRI. These patients were subsequently referred for a 7 T MRI to enable further characterization of the ambiguous vascular finding. This retrospective study was approved by the local ethics committee. All patients were informed about the MRI-related risks before the examinations as a part of the routine procedure and signed a general consent form.

All patients were scanned on a clinically approved 7 T whole-body MRI scanner (MAGNETOM Terra, clinical mode, Siemens Healthcare, Erlangen, Germany) equipped with a 1-channel transmit and 32-channel receive head coil (Nova Medical, Wilmington, MA, USA) and on a 3 T scanner (MAGNETOM Prisma, Siemens Healthcare) with a 32-channel head coil. The 7 T MRI protocol included the following sequences: sagittal T1 MPRAGE (Magnetization Prepared—RApid Gradient Echo) 0.6 × 0.6 × 0.6 mm^3^, axial T2 spin echo (SE) 0.3 × 0.3 × 1.5 mm^3^, axial SWI 0.2 × 0.2 × 1.2 mm^3^, axial arterial time-of-flight (aToF) angiography 0.14 × 0.14 × 0.25 mm^3^ (interpolated, acquired: 0.36 × 0.28 × 0.50 mm^3^) and sagittal T1 sampling perfection with application optimized contrast using different flip angle evolution (sampling perfection with application optimized contrasts using different flip angle evolution, SPACE) 0.5 × 0.5 × 0.5 mm^3^. The 3 T MRI procedure adhered to a standardized contrast-enhanced protocol (including aToF angiography 0.50 × 0.50 × 0.50 mm^3^, vessel wall imaging with axial T1 SPACE 0.50 × 0.50 × 0.50 mm^3^ precontrast and postcontrast and 2D axial T1 SE 2.0 mm slice thickness, contrast-enhanced angiography with bolus timing combined applications to reduce exposure (CARE, bolus, 0.1 mmol/kg, 1.5 ml/s, 0.60 × 0.60 × 0.60 mm^3^)).

### Image Analysis

Reading was performed independently by three neuroradiologists (cumulative level of experience in radiology and neuroradiology: R1, 5 years, resident; R2, 11 years, senior physician; R3, 15 years, senior physician). Readers were blinded to any clinical information. Brain MRI of all patients was presented and reviewed on a PACS station (IDS7, version 23.1, Sectra, Linkoping, Sweden). The imaging data presentation was standardized and stored in a uniform layout comprising all acquired sequences in the respective examination (i.e. including both, aToF and contrast-enhanced angiography at 3 T). Multiplanar reconstruction (MPR), maximum intensity projection (MIP) and 3D rendering provided by the PACS system were used. A short educational module was completed by each reader before image interpretation. The readers were instructed to perform their assessment of all variables for each patient and report them in a standardized spreadsheet based on a predefined written protocol.

The reading order was standardized: an initial review of 3 T imaging studies was performed before reviewing the 7 T imaging studies after a minimum delay of 2 weeks. No randomization was performed in order to avoid potential bias due to the expected information gain if 7 T was followed by 3 T. Blinding to the field strength was not feasible due to the visual characteristics of the images. The following variables were reported: presence of an UIA (yes/no) and level of diagnostic certainty: 1 (poor), 2 (fair), 3 (good) and 4 (excellent). If a UIA was present, the anatomic location, shape (saccular versus fusiform), presence of a bleb, and maximum diameter of the aneurysm (in mm) was recorded. If more than one aneurysm was detected, the same variables were reported for each aneurysm separately.

### Statistical Analysis

Statistical analysis was performed using Stata (StataCorp. 2021. Stata Statistical Software: Release 17. College Station, TX: StataCorp LLC, USA). Descriptive analysis was performed using frequencies and percentages for categorical variables and mean (± standard deviation, SD) or median (interquartile range, IQR) for continuous variables. Fisher’s exact test or Pearson’s χ^2^-test was used for categorical variables, as appropriate, and the Wilcoxon rank-sum test was used to compare continuous variables. The results of all three readers were compared. In the case of dichotomous variables, a qualitative finding was considered positive if two or all three readers scored it as positive. In the analysis of diagnostic certainty, when all three readers disagreed the score of the most experienced reader was the one taken into consideration.

Interrater and intrarater reliability for categorical data was determined by using Cohen/Conger’s’ Kappa, Gwet’s AC, and percentage of agreement. Agreement was defined as almost perfect (κ or interclass correlation coefficient, ICC > 0.80), substantial (κ or ICC = 0.61–0.80), moderate (κ or ICC = 0.41–0.60), fair (κ or ICC = 0.21–0.40), or poor (κ or ICC ≤ 0.20). Presence of an aneurysm assessed by each rater was also compared with the final assessment by the expert neurovascular board derived from the clinical history and taking both the 7 T and 3 T findings into consideration.

## Results

In total, 53 patients with equivocal cerebrovascular findings on lower field strength MRI referred for a 7 T MRI examination were included, resulting in a total of 106 MRI examinations. The median patient age at examination was 56 years (range 16–80 years), with 18 male (34%) and 35 female patients (66%). No patient had a history of a hemorrhage due to aneurysm rupture.

**Fig. 1:**
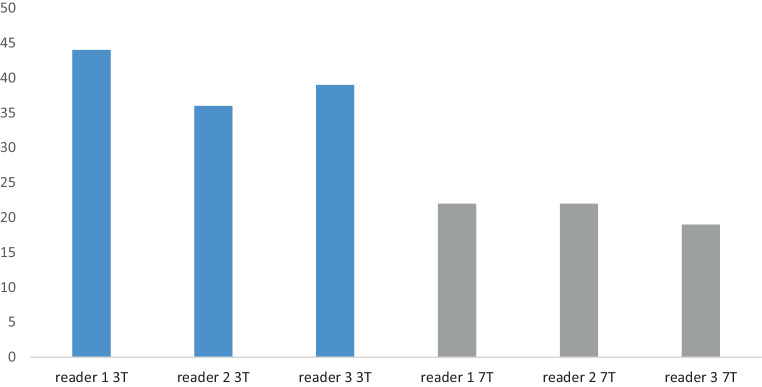
Comparison of the numbers of patients with suspected UIAs determined by readers 1, 2 and 3. 3 T data in *blue*, 7 T data in *grey*

On 3 T MRI, the 3 readers identified imaging correlates of suspected UIAs in 44 (83%), 36 (68%) and 39 (74%) patients, respectively. On 7 T MRI, the respective imaging correlates of suspected UIAs declined to 22 (42%), 22 (42%) and 19 (36%) patients, respectively (Fig. 1).

Aneurysms were suspected in 42 patients examined at 3 T and in 23 patients examined at 7 T (rate difference 36%, 95% CI, 19–53%, *p*-value < 0.001, Supplementary Information: Table 1A and Table 1B for each individual reader, and Fig. 1)**.** The maximum diameter of suspected UIAs was smaller at 3 T (mean 2.9 mm, SD 2.3 mm) than 7 T (mean 4.1 mm, SD 3.1 mm; median 2.2 mm, IQR 1.6–3.0 mm and 2.8 mm, IQR 2.2–4.6 mm, respectively, difference −1.2; 95% confidence interval  −2.5 to 0.2, *p*-value < 0.05; UIA diameters from all three readers are displayed in Supplementary Information, Table 2). The UIA locations in aggregated data include only cases where at least 2 readers agreed on the presence of a UIA (cases about which all readers disagreed were excluded from this specific analysis). Major disagreement between findings at the different field strengths was observed in the A1/ACOM complex (Table 1*.* Data for all individual readers are shown in the Supplementary Information, Table 3). Most UIAs had a saccular shape at both field strengths (Supplementary Information, Table 4).

**Table 1:** Comparison of findings at the different field strengths

	3 T	7 T
	n (%)	n (%)
A2	3 (5.7)	0 (0.00)
A1/ACOM	9 (17)	2 (3.8)
BA	4 (7.5)	2 (3.8)
ICA	9 (17)	8 (15)
M1	7 (13)	5 (9.4)
M2	3 (5.7)	2 (3.8)
P1	3 (5.7)	1 (1.9)
PCOM	1 (1.9)	0 (0.00)

*ACOM* anterior communicating artery, *BA* basilar artery, *ICA* internal carotid artery, *PCOM* posterior communicating artery

In general, interrater agreement among the three readers on the presence of an aneurysm on 3 T MRI was lower than that for 7 T MRI. Interrater agreement for 3 T MRI was 0.786 (95% CI 0.700–0.873; 0.438 Cohen/Conger’s kappa, 95% CI 0.222–0.654; 0.657 Gwet’s AC, 95% CI 0.492–0.822) and for 7 T 0.925 (95% CI 0.866–0.983; 0.842 Cohen/Conger’s Kappa, 95% CI 0.723–0.962; 0.855 Gwet’s AC, 95% CI 0.739–0.972). Each pair of 3 T/7 T examinations was analyzed to assess between-scan intrarater variability for each reader independently. This revealed low intrarater agreement between 3 and 7 T examinations (Table 2)*.*

**Table 2:** Between-scan intrarater variability for each reader

	Percent agreement	Cohen/Conger’s kappa	Gwet’s AC
Reader 1	0.585 (0.448–0.722)^a^	0.254 (0.089–0.418)	0.217 (−0.071–0.505)
Reader 2	0.736 (0.613–0.859)	0.502 (0.301–0.703)	0.476 (0.231–0.722)
Reader 3	0.623 (0.488–0.758)	0.334 (0.157–0.512)	0.252 (−0.020–0.524)

^a^numbers in parentheses represent 95% Confidence Interval (CI)

Diagnostic certainty of the readers (on a scale from 1–4) was greater at 7 T than at 3 T to for all readers and showed lower variability at 7 T (Table 3; Supplementary Information, Fig. 2. Variable referenced to reader 3 as the most experienced; data for each individual reader in the Supplementary Information, Table 5). The level of agreement of diagnostic certainty aggregated for all three readers was lower for 3 T MRI than for 7 T MRI *(*Table 4*)*.

**Table 3:** Diagnostic certainty of the readers rated on a scale of 1–4

	3 T (*n* = 53)	7 T (*n* = 53)	Rate difference (95% confidence interval)
	n (%)	n (%)	
Level of diagnostic certainty (aggregated over 3 readers)			
Poor (1)	–	–	N/A
Fair (2)	5 (9.4%)	0 (0.00%)	0.09 (0.02–0.17)
Good (3)	25 (47%)	2 (3.8%)	0.43 (0.29–0.58)
Excellent (4)	23 (43%)	51 (96%)	−0.53 (−0.67 to −0.39)

**Table 4:** Level of agreement of diagnostic certainty at 3 T and 7 T aggregated for all three readers

	Interrater agreement between readers for 3 T		Interrater agreement between readers for 7 T	
	Percent agreement	Gwet’s AC	Percent agreement	Gwet’s AC
Level of diagnostic certainty (aggregated)	0.871 (0.842–0.900)	0.687 (0.597–0.777)	0.920 (0.884–0.957)	0.900 (0.847–0.954)

In 22 (reader 1), 15 (reader 2), and 18 (reader 3) patients aneurysms were detected in 3 T and were not confirmed in the expert neurovascular board assessment (false positive, examples in Supplementary Information, Fig. 3). In none (reader 1) or one patient (reader 2 and reader 3), aneurysms were detected in 7 T and were not confirmed in the expert neurovascular board assessment (false positive).

## Discussion

This retrospective analysis demonstrates a significantly higher interrater agreement and improved diagnostic certainty when small intracranial aneurysms are visualized on 7 T MRI compared to 3 T. The readers overestimated the presence of an aneurysm more often on 3 T MRI than on 7 T MRI, indicating a much higher risk of false positive findings when using 3 T MRI. The most prevalent changes from UIA at 3 T to anatomical variant at 7 T were infundibular branches and perforating arteries interpreted initially as aneurysms. Less frequent findings were small fenestrations and plexiform variants of the ACOM. Moreover, the increase in size of the postulated UIAs on 7 T MRI compared to the 3 T reading suggests that smaller findings interpreted as UIAs at 3 T tend to be interpreted as normal vessels at 7 T. The most frequent location for false positive findings at 3 T was ACOM/A1. This could be explained by the high anatomical variability in this region.

This study has limitations that need to be considered when interpreting the results. The gold standard of digital subtraction angiography (DSA) was omitted in accordance with the guidelines for UIA depiction. In addition, performing an invasive procedure would not necessarily have been justifiable in this cohort of patients. Although the absence of DSA data is a limitation, a strong correlation between 7 T aToF and DSA has been shown previously, with some studies reporting sensitivity rates comparable to those of DSA [10, 11]. A further limitation of the present study is the lack of blinding to the field strength, which was not feasible due to the obvious differences in image quality. Although we consider the cohort under investigation with equivocal findings on 3 T MRI to be a limited subgroup of patients, this reflects a relevant clinical question for routine imaging with consequences for individual patient management. In particular, small findings (< 5 mm) in cerebral vessels may remain ambiguous: up to 18% of findings on 3 T 3D aToF may be unclear and in the remaining 82% regarded as clear, also false positive and negative rates up to 6% and 2%, respectively, have been reported [1, 12]. The presented work has had an influence on the clinical practice at our institution (Institute of Diagnostic and Interventional Neuroradiology, Bern University Hospital). Currently patients with ambiguous intracranial vascular findings and suspicion of small aneurysms at lower field strength MRI are referred to a 7 T imaging for clarification. In the past, such patients underwent follow-up examinations at 1.5 or 3 T or, in selected cases, DSA. Moreover, a dedicated cost-effectiveness analysis might be worth conducting, as 7 T MRI may reduce the need to perform unnecessary follow-up MRIs or invasive work-ups (i.e. DSA), and thus decrease the burden on the healthcare system.

In conclusion, 7 T MRI is a powerful diagnostic tool for differentiating true UIAs from normal anatomical variants of intracranial arteries such as infundibula or vascular loops. In a selected patient cohort, clinical implementation of 7 T MRI may help to establish the definitive diagnosis and thus have a beneficial impact on patient management.

### Supplementary Information

Supplementary information displaying additional results: Supplementary Tables 1 to 5. Supplementary Figures 1 to 3
