# Supplementary material for: Diagnosis of Small Unruptured Intracranial Aneurysms: Comparison of 7 T versus 3 T MRI
Source: Clin Neuroradiol. 2023 Mar 31;34(1):45–9. doi: 10.1007/s00062-023-01282-2 (PMC10881608; doi:10.1007/s00062-023-01282-2)
Supplement: Supplementary file 1 — Supplementary information displaying additional results: Supplementary Tables 1 to 5. Supplementary Figures 1 to 3 [file 62_2023_1282_MOESM1_ESM.docx]

**Supplementary Information**

**Table 1A: Suspected unruptured intracranial aneurysms**

|  | 3T (N = 53) | 7T (N = 53) | | Rate difference  (95% CI) |
| --- | --- | --- | --- | --- |
|  | n (%) | n (%) |  | |
| Aneurysm present (aggregated for all 3 readers) | 42 (79) | 23 (43) | 36% (19 to 53) | |

**Table 1B: Suspected unruptured intracranial aneurysms, individual readers**

|  | 3T (N = 53) | 7T (N = 53) | Rate difference (95% CI) | P-value * |
| --- | --- | --- | --- | --- |
|  | n (%) | n (%) |  |  |
| Aneurysm present (aggregated) | 42 (79) | 23 (43) | 0.36 (0.19 to 0.53) | <0.001 |
| Aneurysm present (R1) | 44 (83) | 22 (42) | 0.42 (0.25 to 0.58) | <0.001 |
| Aneurysm present (R2) | 36 (68) | 22 (42) | 0.26 (0.08 to 0.45) | 0.006 |
| Aneurysm present (R3) | 39 (74) | 19 (36) | 0.38 (0.20 to 0.55) | <0.001 |

* Pearson’s chi-squared test

**Figure 1 :** Rate difference 3T versus 7T

**Table 2: Diameter of suspected unruptured intracranial aneurysms reported by each reader**

|  | 3T | 7T |
| --- | --- | --- |
|  | Mean (SD) in mm | Mean (SD) in mm |
| Max. diameter (reader 1) | 2.9 (2.4) | 4.1 (3.0) |
| Max. diameter (reader 2) | 3.5 (2.8) | 4.6 (3.3) |
| Max. diameter (reader 3) | 2.8 (2.0) | 3.5 (3.0) |

**Table 3: Unruptured intracranial aneurysms locations reported by each reader**

|  | 3T | 7T |
| --- | --- | --- |
|  | n (%) | n (%) |
| Location (R1) |  |  |
| A1 | 1 (1.9) | 0 (0.00) |
| A2 | 4 (7.5) | 0 (0.00) |
| ACOM | 10 (19) | 2 (3.8) |
| BA | 4 (7.5) | 2 (3.8) |
| ICA | 11 (21) | 9 (17) |
| M1 | 7 (13) | 8 (15) |
| M2 | 3 (5.7) | 0 (0.00) |
| P1 | 4 (7.5) | 1 (1.9) |
| Location (R2) |  |  |
| A1 | 1 (1.9) | 0 (0.00) |
| A2 | 3 (5.7) | 0 (0.00) |
| ACOM | 7 (13) | 2 (3.8) |
| BA | 4 (7.5) | 2 (3.8) |
| ICA | 7 (13) | 7 (13) |
| M1 | 3 (5.7) | 2 (3.8) |
| M2 | 6 (11) | 6 (11) |
| P1 | 2 (3.8) | 1 (1.9) |
| PCOM | 3 (5.7) | 2 (3.8) |
| Location (R3) |  |  |
| A2 | 3 (5.7%) | 1 (1.9) |
| ACOM | 8 (15) | 2 (3.8) |
| BA | 3 (5.7) | 1 (1.9) |
| ICA | 8 (15) | 6 (11) |
| M1 | 8 (15) | 5 (9.4) |
| M2 | 4 (7.5) | 3 (5.7) |
| P1 | 4 (7.5) | 1 (1.9) |
| PCOM | 1 (1.9) | 0 (0.00) |

ACOM, anterior communicating artery; BA, basilar artery; ICA internal carotid artery; PCOM, posterior communicating artery.

**Table 4: Shape of unruptured intracranial aneurysms**

|  | 3T (N = 53) | 7T (N = 53) |
| --- | --- | --- |
|  | n (%) | n (%) |
| Shape (R1) |  |  |
| saccular | 40 (75) | 17 (32) |
| fusiform | 4 (7.5) | 4 (7.5) |
| Shape (R2) |  |  |
| saccular | 32 (60) | 19 (36) |
| fusiform | 4 (7.5) | 3 (5.7) |
| Shape (R3) |  |  |
| saccular | 38 (72) | 19 (36) |
| fusiform | 1 (1.9) | 0 (0) |
| Multilobar/daughter aneurysms (R1) |  |  |
| no | 41 (77) | 18 (34) |
| yes | 3 (5.7) | 4 (7.5) |
| Multilobar/daughter aneurysms (R2) |  |  |
| no | 33 (62) | 17 (32) |
| yes | 3 (5.7) | 5 (9.4) |
| Multilobar/daughter aneurysms (R3) |  |  |
| no | 37 (70) | 16 (30) |
| yes | 2 (3.8) | 3 (5.7) |
| Multilobar/daughter aneurysms (aggregated) |  |  |
| no | 45 (85) | 19 (36) |
| yes | 2 (3.8) | 4 (7.5) |

**Table 5: Diagnostic confidence for each individual reader**

|  | 3T (N = 53) | 7T (N = 53) | Rate difference (95% CI) | P-value |
| --- | --- | --- | --- | --- |
|  | n (%) | n (%) |  |  |
| Level of diagnostic confidence (R1) |  |  |  | <0.001 |
| poor | 3 (5.7) | 0 (0.00) | 0.06 (-0.01 to 0.12) |  |
| fair | 10 (19) | 1 (1.9) | 0.17 (0.06 to 0.28) |  |
| good | 19 (36) | 15 (28) | 0.08 (-0.10 to 0.25) |  |
| excellent | 21 (40) | 37 (70) | -0.30 (-0.48 to -0.12) |  |
| Level of diagnostic confidence (R2) |  |  |  | <0.001 |
| fair | 13 (25) | 0 (0.00) | 0.25 (0.13 to 0.36) |  |
| good | 23 (43) | 0 (0.00) | 0.43 (0.30 to 0.57) |  |
| excellent | 17 (32) | 53 (100) | -0.68 (-0.80 to -0.55) |  |
| Level of diagnostic confidence (R3) |  |  |  | <0.001 |
| fair | 4 (7.5) | 0 (0.00) | 0.08 (0.00 to 0.15) |  |
| good | 20 (38) | 3 (5.7) | 0.32 (0.18 to 0.47) |  |
| excellent | 29 (55) | 50 (94) | -0.40 (-0.54 to -0.25) |  |

**Figure 2:** Diagnostic confidence score on a scale of 1 to 4 for each individual reader. From left to right: 3T data in blue (reader 1, 2 and 3), 7T data in grey (reader 1, 2 and 3).


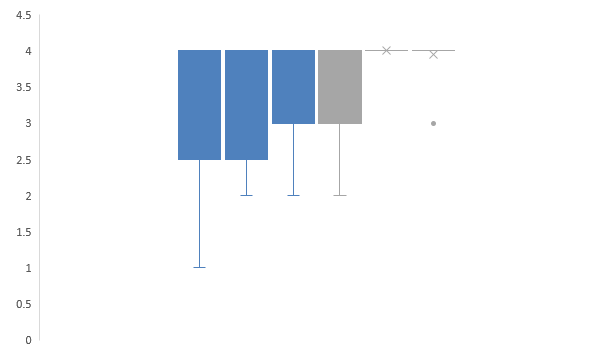


**Figure 3:** Two representative examples.

Panels A-C display an ambiguous finding in the left A2 Segment on 3T MRI and panels D-F display the same finding on 7T (from top to bottom, axial ToF, zoom on axial ToF and 3D reconstruction. Blue arrows indicate the findings).

Panels G-I display an ambiguous finding in the Acom-Complex on 3T MRI and panels J-L display the finding on 7T (from top to bottom, axial ToF, zoom on axial ToF and 3D reconstruction. Blue arrows indicate the findings).

| 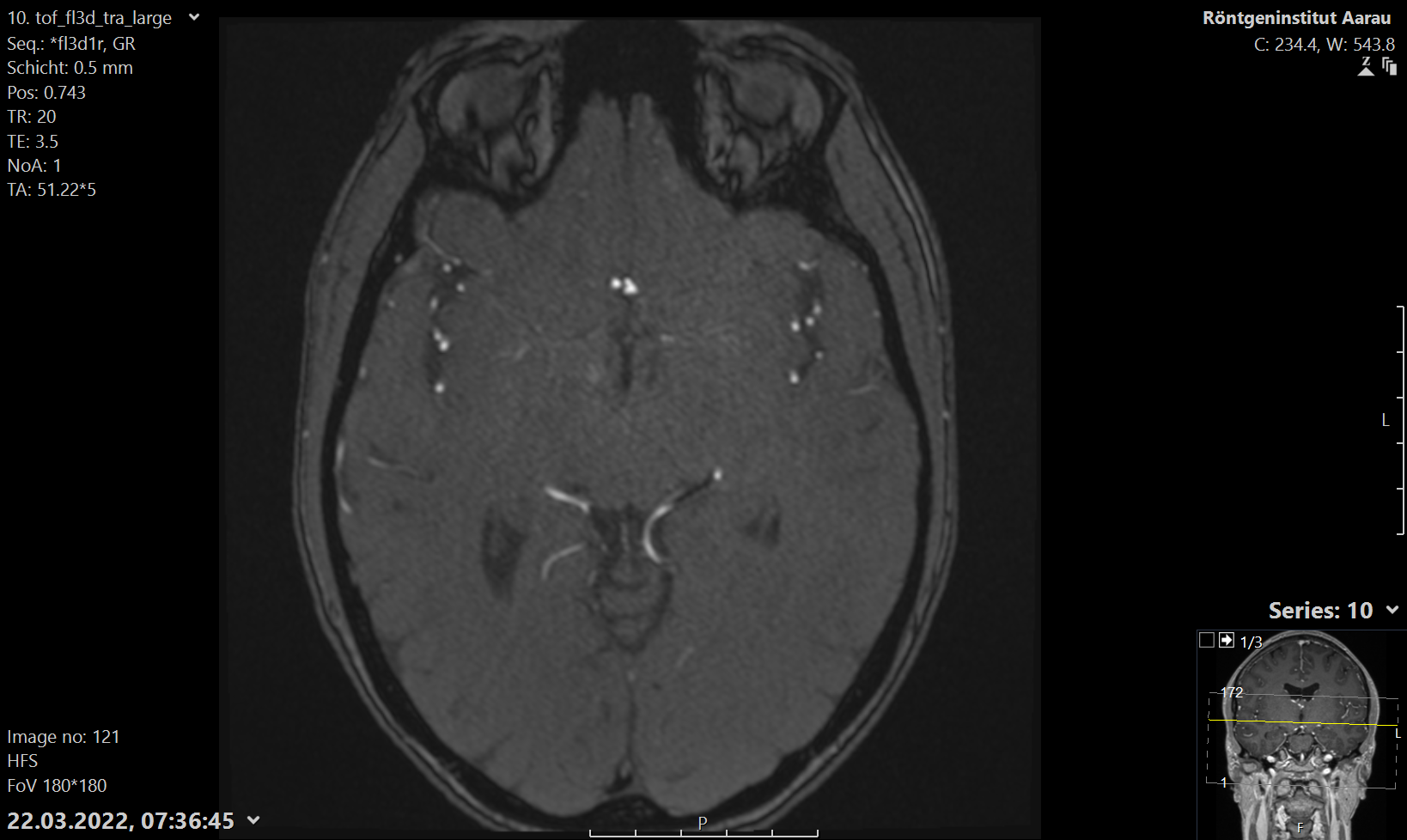  A | 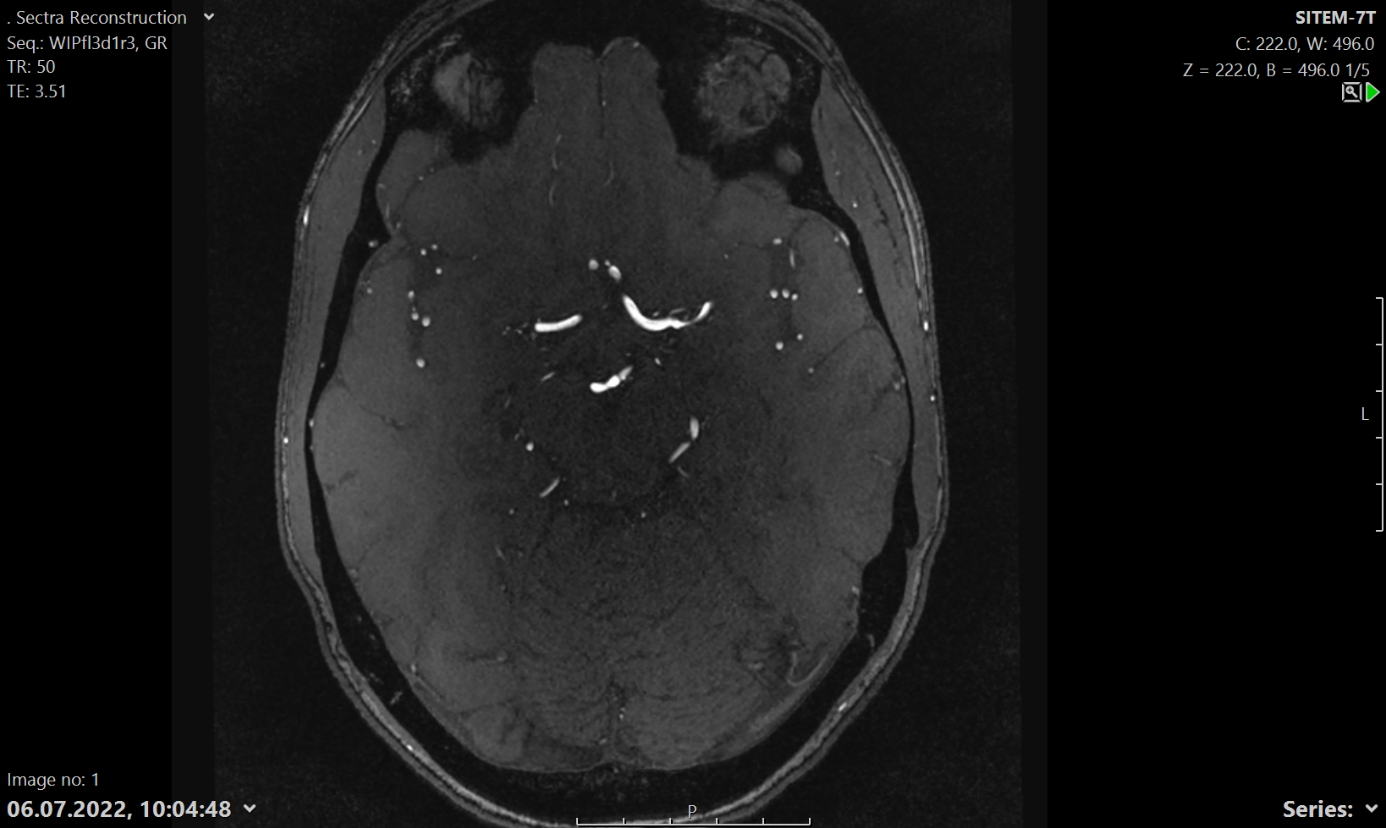  E  D |
| --- | --- |
| 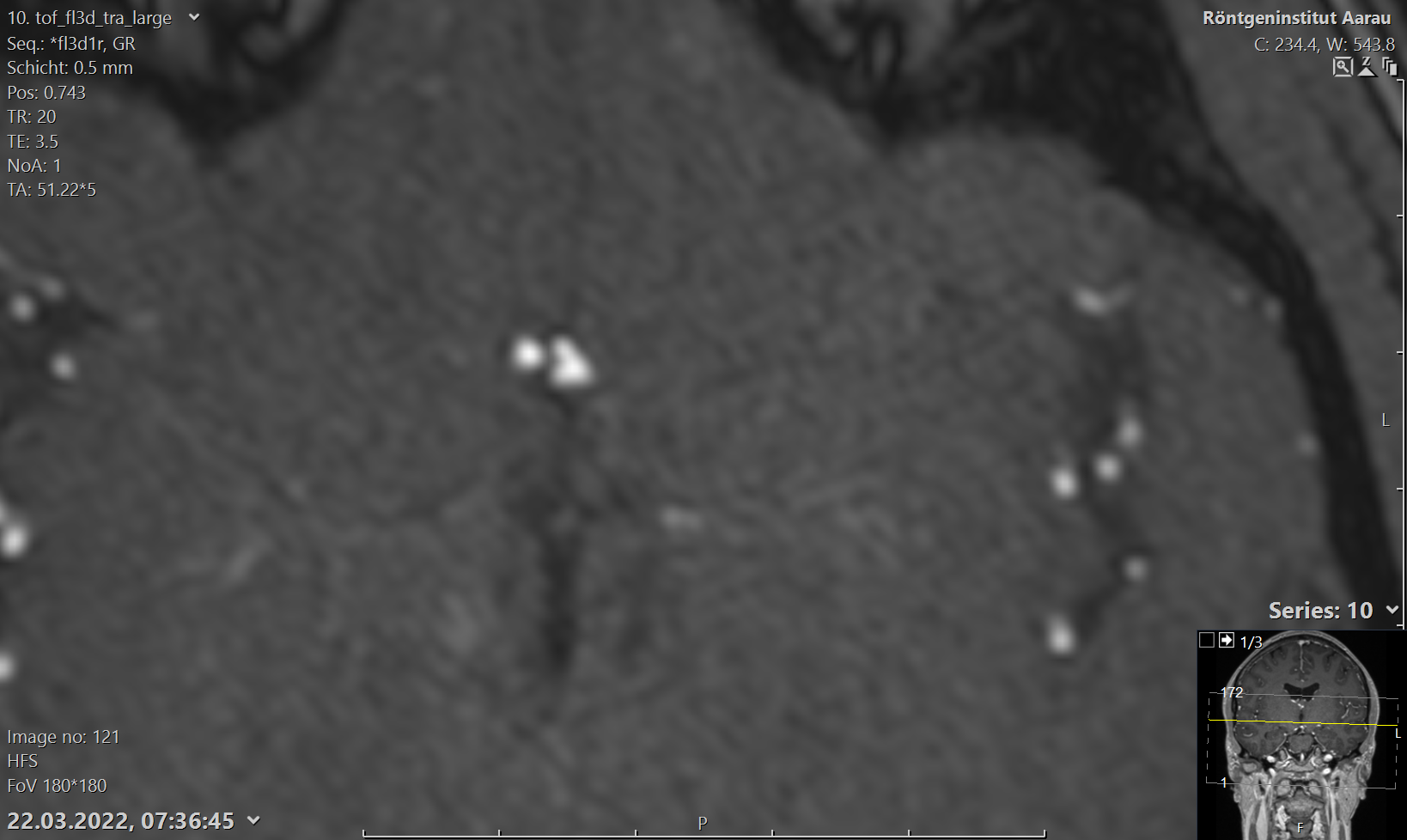  B | 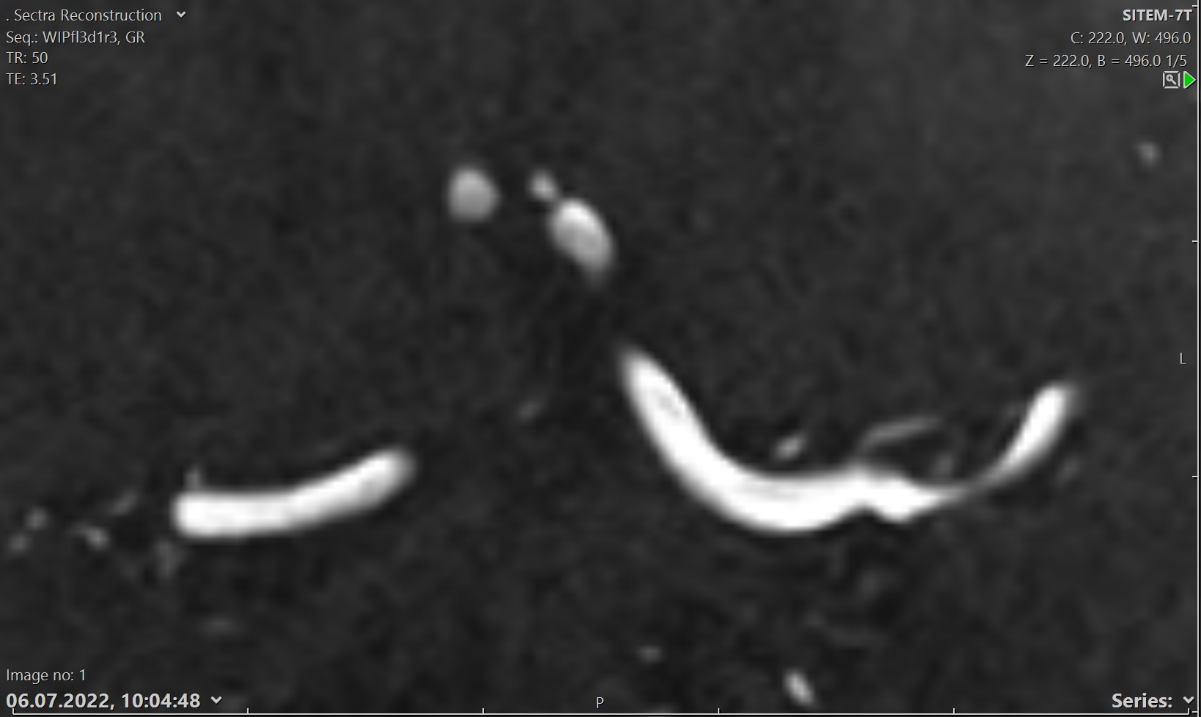 |
| 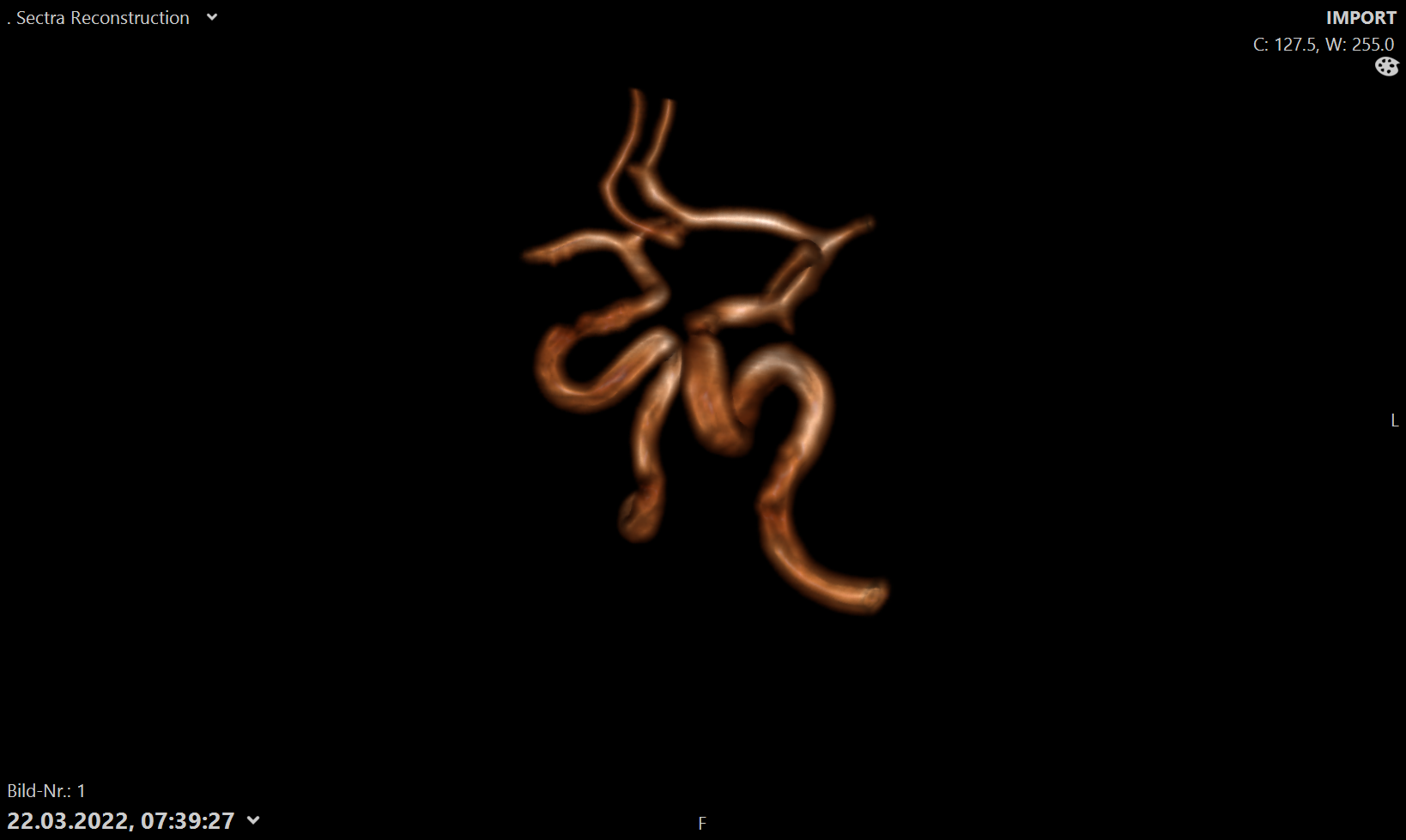  C | 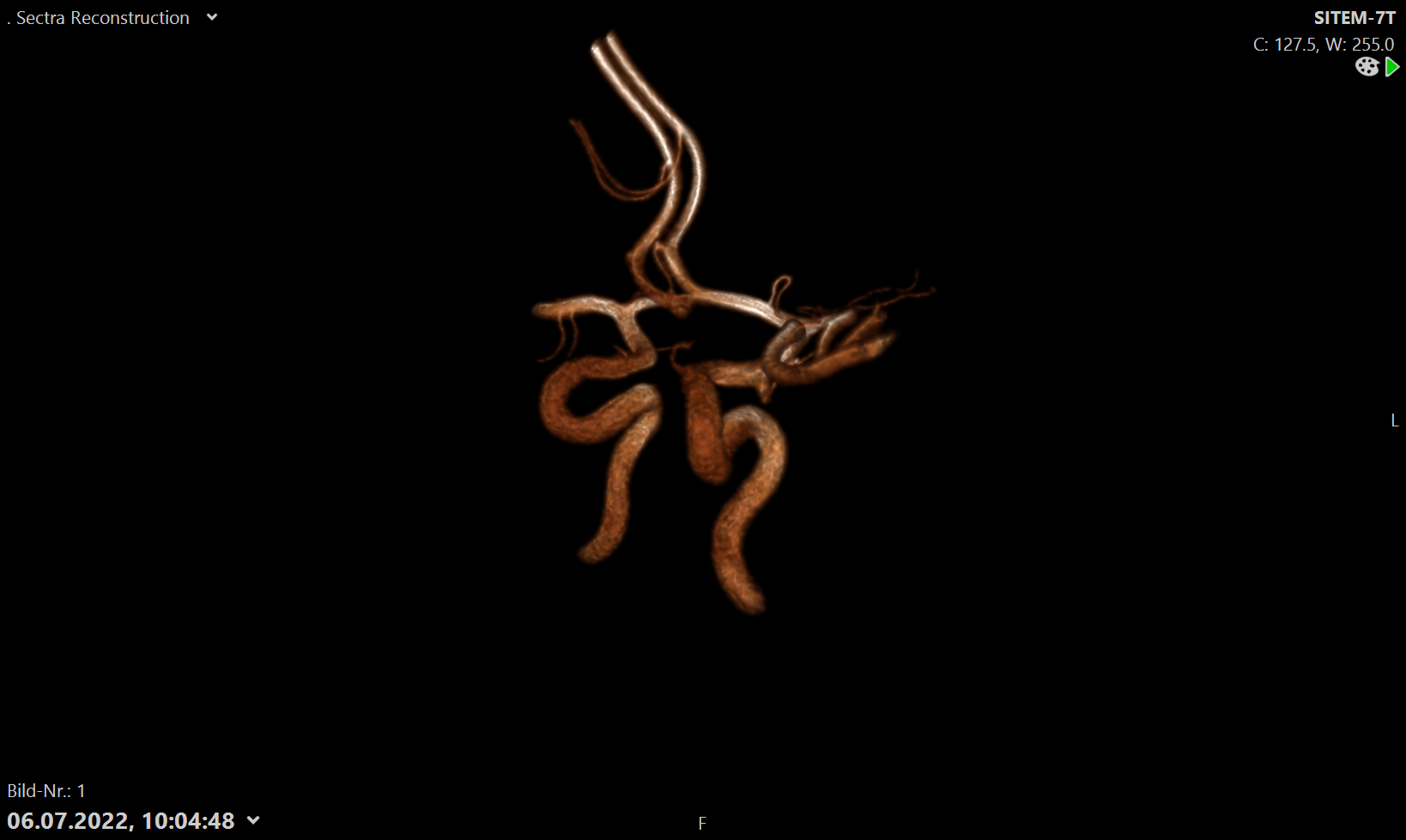  F |

| 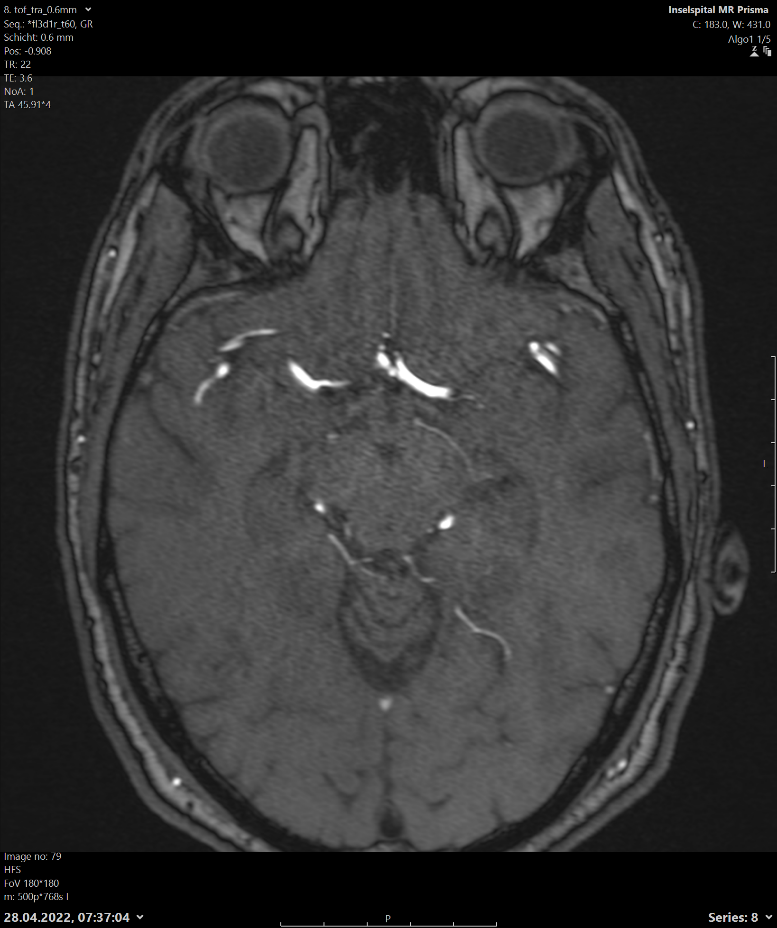  G | 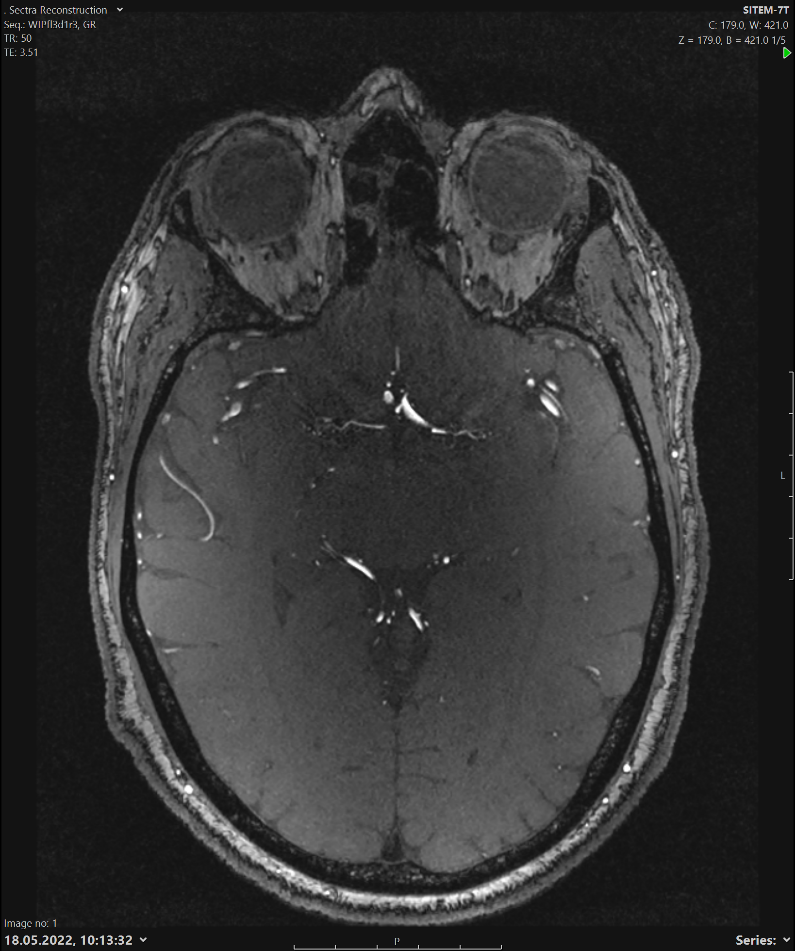  J |
| --- | --- |
| 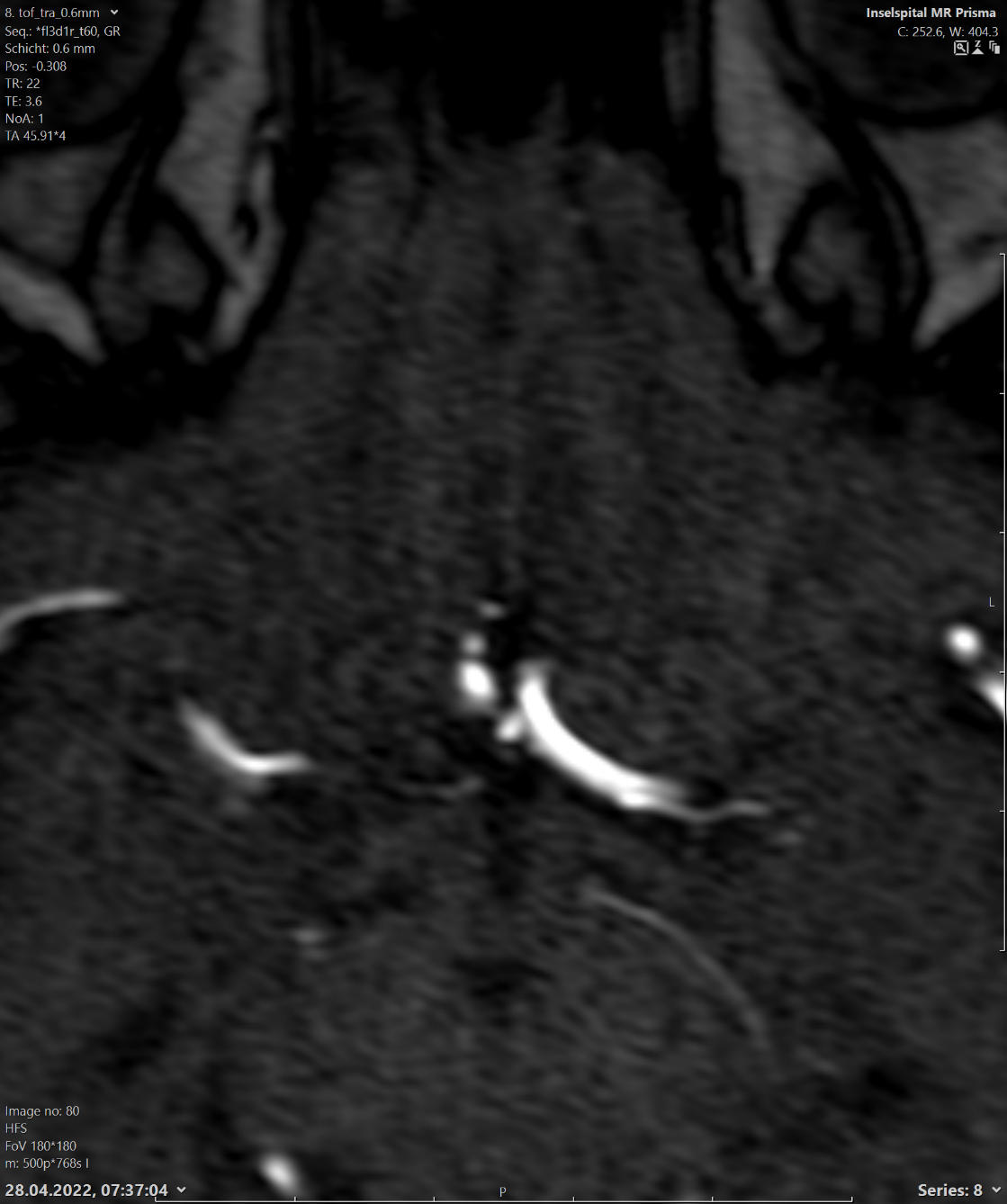  H | 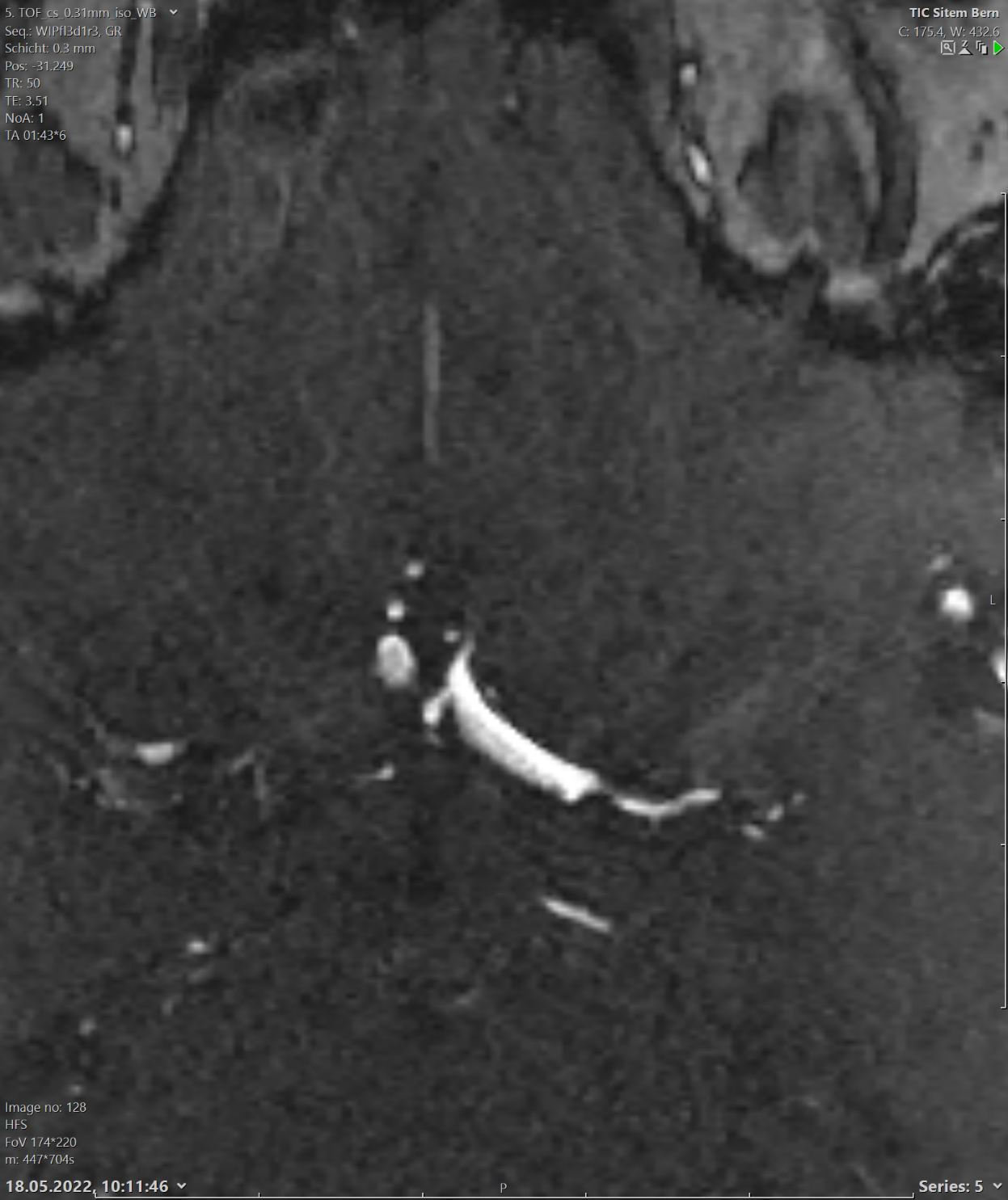  K |
| 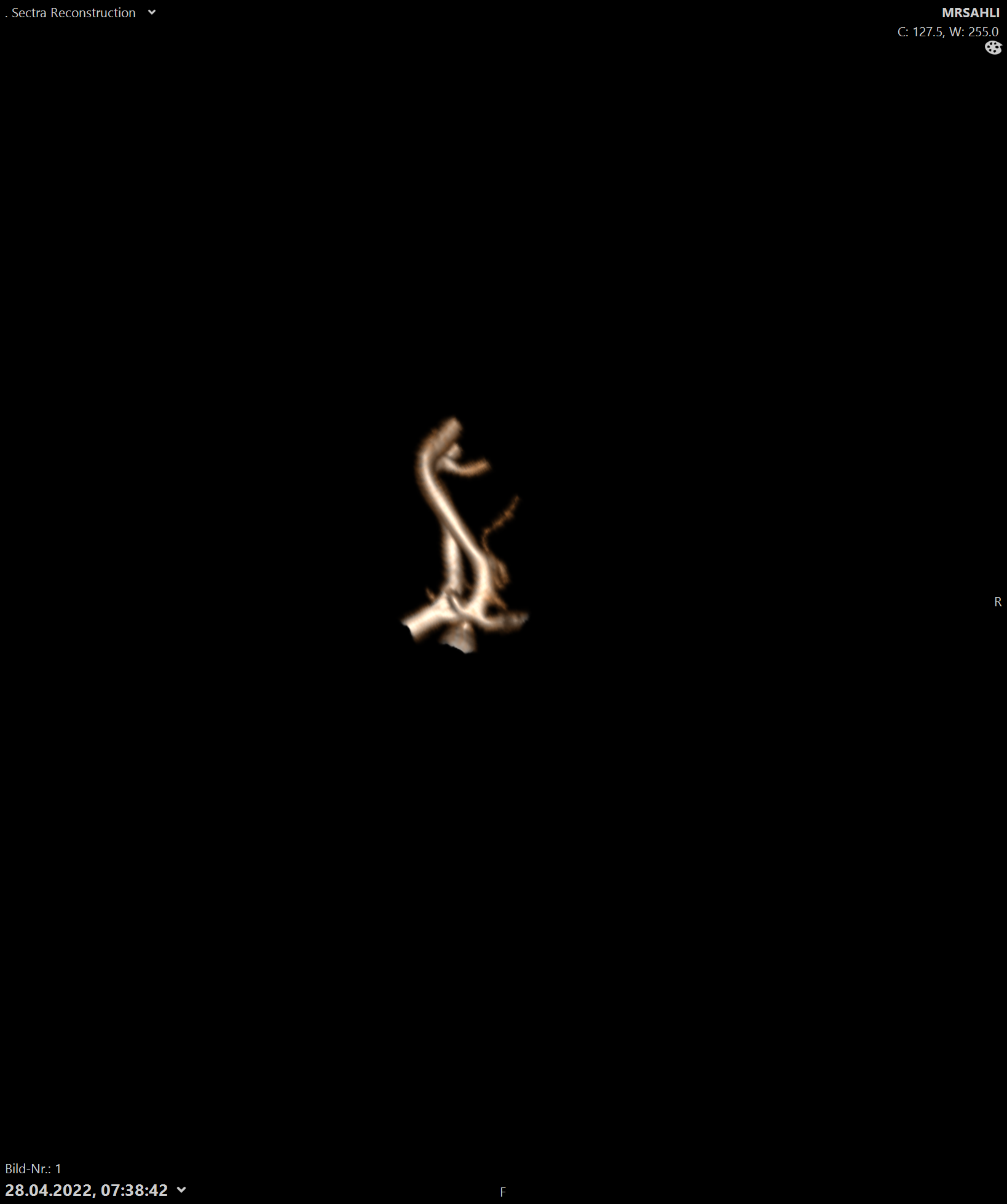  I | 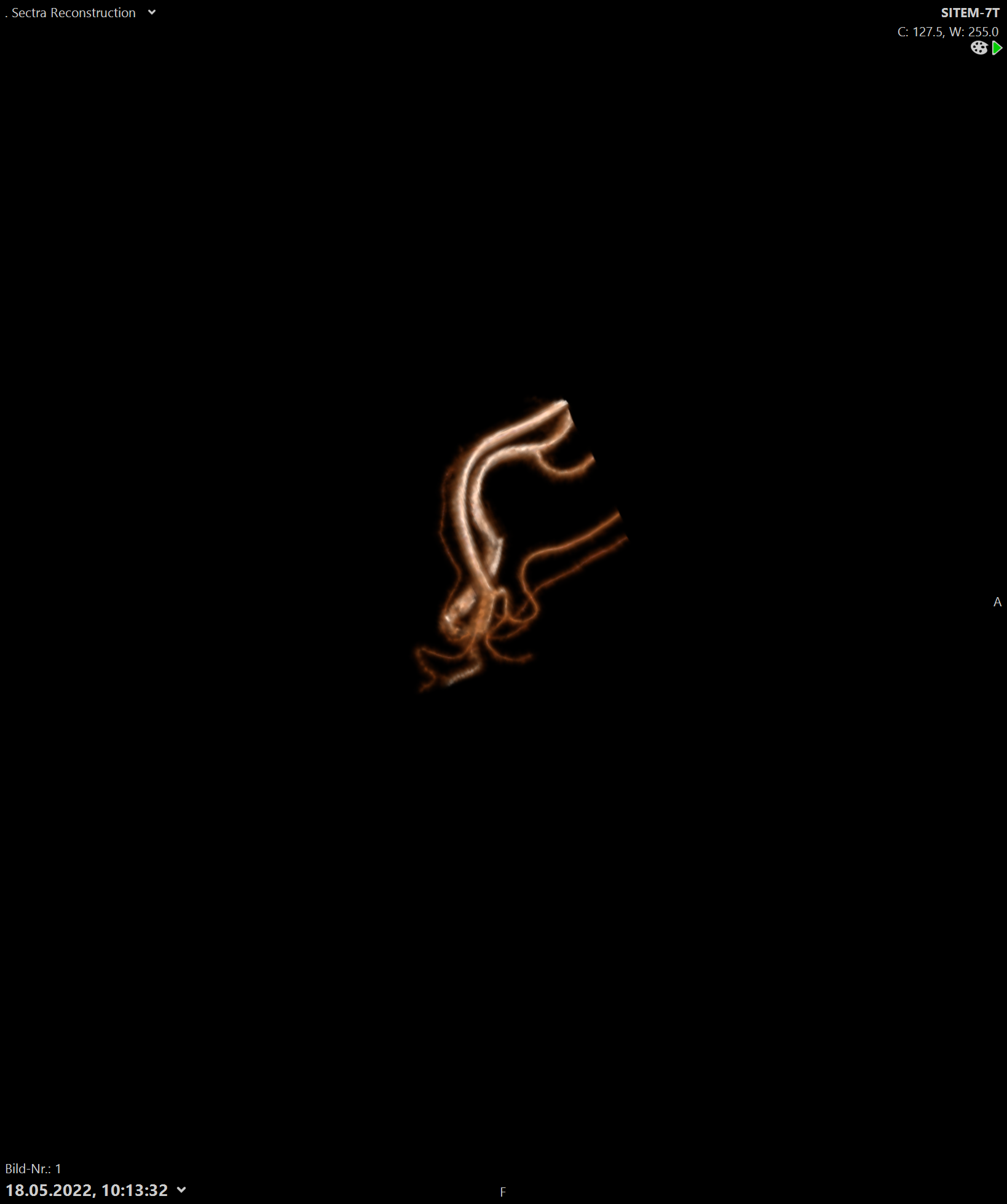  L |
